# High-throughput sequencing data and antibiotic resistance mechanisms of soil microbial communities in non-irrigated and irrigated soils with raw sewage in African cities

**DOI:** 10.1016/j.dib.2019.104638

**Published:** 2019-10-15

**Authors:** B.P. Bougnom, S. Thiele-Bruhn, V. Ricci, C. Zongo, L.J.V. Piddock

**Affiliations:** a Institute of Microbiology and Infection, University of Birmingham, B15 2TT, UK; b Department of Microbiology, Faculty of Science, University of Yaounde 1, P.O. Box 812, Yaounde, Cameroon; c Department of Soil Science, University of Trier, D-54286, Trier, Germany; d Department of Biochemistry and Microbiology, University Ouaga I Pr Joseph KI-ZERBO, 03 BP 7021, Ouagadougou 03, Burkina Faso

**Keywords:** Wastewater irrigation, Urban agriculture, Antibiotic resistance, Metagenomics, Africa

## Abstract

High-throughput sequencing data of soil microbial communities in non-irrigated and irrigated soils with raw sewage in African cities are presented in this report. These data were collected to study the potential of wastewater use in urban agriculture to disseminate bacterial resistance in soil. Soil samples were collected in three cities in two African countries. Each city had two sectors (irrigated and non-irrigated). After collection, biomass samples were purified, DNA from soil was extracted, quantified and sequenced using multiplex Illumina high-throughput sequencing. The sequence count of the six metagenome datasets ranges from 3,258,523,350 bp to 4,120,454,250 bp; the mean sequence length post quality control average was 149 ± 3 bp. The mechanisms of resistance encoded by the identified antibiotic resistance genes (ARGs) in the metagenomic data were dominated by antibiotic inactivation enzymes (64.7% and 71.9%), followed by antibiotic target replacement (14.7% and 12.5%), antibiotic target protection (11.8% and 9.4%) and efflux pumps (6.3% and 8.8%) in bacterial DNA isolated from irrigated and non-irrigated fields, respectively. The datasets will be useful for the scientific community working in the area of bacterial resistance dissemination from the environment. They can be used for further understanding of bacterial drug-resistance gene prevalence and acquisition in wastewater irrigated soils. The data reported herein was used for the article, titled “Raw wastewater irrigation for urban agriculture in three African cities increases the abundance of transferable antibiotic resistance genes in soil, including those encoding Extended spectrum β-lactamase (ESBLs)” Bougnom et al. (2020) [1].

**Table:** Specifications Table

Subject	Biology
Specific subject area	Microbiology and genomics
Type of data	Figure and Shotgun metagenomic sequencing data.
How data were acquired	Soil samples were collected and purified. Soil DNA was extracted, and multiplex high-throughput sequencing was conducted according to Illumina sequencing protocols for DNA-seq.
Data format	Raw metagenomic data, analyzed and deposited.
Parameters for data collection	Soil samples were collected in three African cities (Ouagadoudou (46°38′ N, 11°29′) in Burkina Faso, Ngaoundere (46°38′ N, 11°29′) and Yaounde (46°38′ N, 11°29′) in Cameroon.). In each city, two sectors were sampled, comprising three agricultural fields that were irrigated with raw wastewater, and three non-irrigated agricultural fields, with comparable soil properties.
Description of data collection	Soil samples were purified, soil DNA was extracted using DNeasy PowerSoil Kit (Qiagen, Germany); quantified using the Quant-iT PicoGreen dsDNA Assay Kit, and the Qubit™ 3.0 Fluorometer (Qubit, Life Technologies, USA); TruSeq DNA Nano gel free library (350 bp insert) was used to prepare the libraries; and multiplex high-throughput sequencing was conducted using Illumina Hiseq4000 platform (Illumina, Inc, USA).
Data source location	The raw metagenomic data have been deposited in MG-RAST server (project IDs: mgm4815682.3; mgm4815683.3; mgm4815684.3; mgm4815685.3; mgm4815686.3; and mgm4815687.3).
Data accessibility	Public repositories Repository name: Mendeley Data (Table 1) Data identification number: https://doi.org/10.17632/db52syhgr8.1 Direct URL to data: https://data.mendeley.com/datasets/db52syhgr8/1 Repository name: MG-RAST server (Raw metagenomic data) Direct URL to data: https://www.mg-rast.org/linkin.cgi?project=mgp87146
Related research article	B. P. Bougnom; S. Thiele*-*Bruhn; V. Ricci; C. Zongo; L.J.V Piddock (2020). Raw wastewater irrigation for urban agriculture in three African cities increases the abundance of transferable antibiotic resistance genes in soil, including those encoding Extended spectrum β-lactamase (ESBLs) Journal: Science of the Total Environment DOI: https://doi.org/10.1016/j.scitotenv.2019.134201

**Table:** 

**Value of the Data** • The data provides insight into the microbial diversity and functional changes after raw sewage irrigation. • The data will be useful for the scientific community working in the area of bacterial drug-resistance gene dissemination in the environment. • The data can be used for further understanding of bacterial drug-resistance acquisition in wastewater irrigated soils. Thus, assessing the public health issue of urban agriculture in low- and middle-income countries.

## 1 Data

**Fig. 1:**
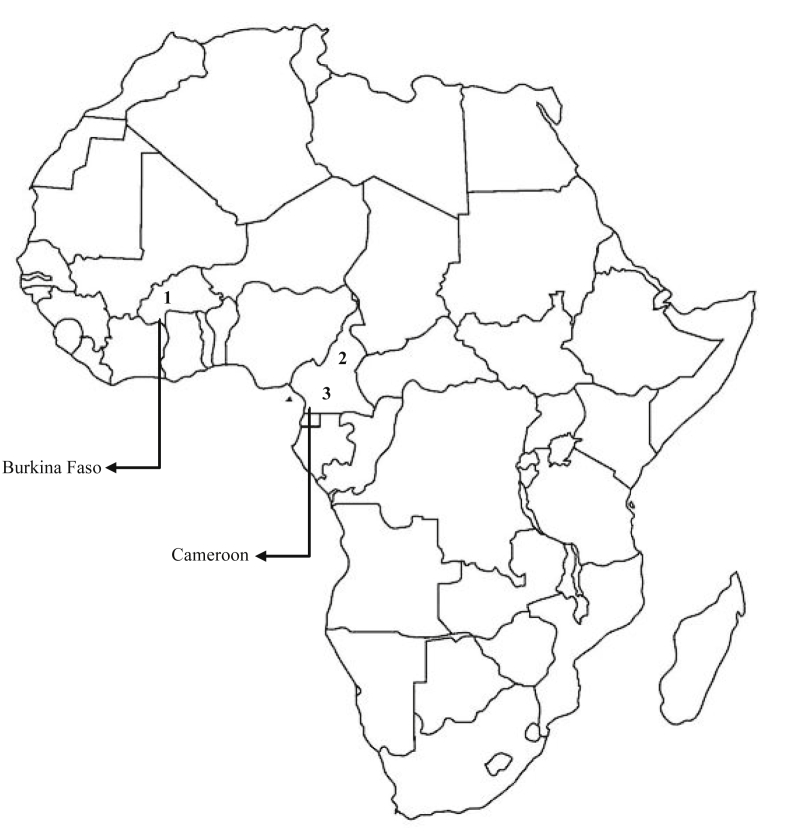
African map showing the investigated countries and cities. 1: Ouagadougou; 2: Ngaoundere; and 3: Yaounde.

**Fig. 2:**
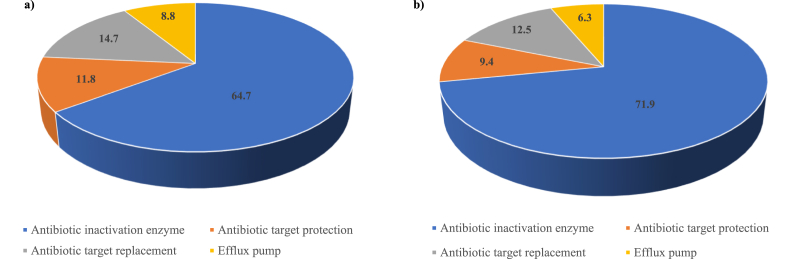
Mechanisms of antibiotic resistance (%) of the antibiotic resistance genes (based on their abundance) derived from the metagenomic reads in (a) irrigated fields and (b) non-irrigated fields (n = 3).

In the present work, we report DNA sequence read metrics of six metagenomic samples from soil obtained from non-irrigated fields (NIR) and their corresponding irrigated fields (IRI) with raw sewage in three cities (Table 1), in two African countries (Fig. 1) [1]. The sequence counts of the metagenome datasets post quality control (QC) ranged from 3,309,468,880 bp to 3,649,105,747 bp and 3,159,665,932 bp to 3,682,552,830 bp in irrigated and non-irrigated fields, respectively. The mean GC content post QC ranged from 60 ± 12% to 65 ± 10% and 62 ± 12% and 66 ± 9% in irrigated and non-irrigated fields, respectively. The mean sequence length post quality control (QC) average was 149 ± 3 bp. The mechanisms of drug-resistance encoded by the identified antibiotic resistance genes (ARGs) in the metagenome data were dominated by antibiotic inactivation enzymes (64.7% and 71.9%), followed by antibiotic target replacement (14.7% and 12.5%), antibiotic target protection (11.8% and 9.4%) and efflux pumps (6.3% and 8.8%) in irrigated and non-irrigated fields, respectively (Fig. 2). The number of ARGs encoding drug-resistance due to antibiotic inactivation enzymes was 6% lower in non-irrigated fields, whereas those encoding the other mechanisms of resistance were 2% higher in irrigated fields. The raw FASTQ metagenomic reads have been deposited in MG-RAST server (project IDs: mgm4815682.3; mgm4815683.3; mgm4815684.3; mgm4815685.3; mgm4815686.3; and mgm4815687.3).

**Table 1:** DNA sequence read metrics of the six metagenomic data from irrigated (IRI) and non-irrigated (NIR) agricultural fields based on MG-RAST annotation.

Metagenome		Sequences count	Sequences count post QC	Mean GC content post QC	Mean sequence length post QC
Irrigated	IRI1	3,792,462,000 bp	3,649,105,747 bp	65 ± 10%	149 ± 3 bp
	IRI2	3,439,886,400 bp	3,309,468,880 bp	62 ± 12%	149 ± 3 bp
	IRI3	3,491,007,600 bp	3,329,257,884 bp	60 ± 12%	149 ± 3 bp
Non-irrigated	NIR1	3,527,491,650 bp	3,394,411,184 bp	63 ± 11%	149 ± 3 bp
	NIR2	3,258,523,350 bp	3,159,665,932 bp	66 ± 9%	149 ± 3 bp
	NIR3	4,120,454,250 bp	3,682,552,830 bp	62 ± 12%	150 ± 3 bp

## 2 Experimental design, materials, and methods

Soil samples were collected in three cities, in two African countries, namely Ouagadougou (46°38′ N, 11°29′) in Burkina Faso, Ngaoundere (46°38′ N, 11°29′) and Yaounde (46°38′ N, 11°29′) in Cameroon (Fig. 1). In each city, there were two sectors comprising three agricultural fields that were irrigated (IRI) with raw wastewater, and as control soils, 500 m away, three non-irrigated agricultural fields (NIR) with comparable soil properties. This gave samples from Ouagadougou (IRI1 and NIR1), Ngaoundere (IRI2 and NIR2), and Yaounde (IRI3 and NIR3). Wastewaters were collected from canals. The canals are natural open-air water drainage canals and collection points of different transects. They receive wastewater from habitations, hospitals, agriculture, markets and slaughterhouses. Salad and tomatoes were the growing plants in the fields. The agricultural fields were approximately 0.2 ha each and watered manually twice per day with watering cans. In each field, 100 g of soil was randomly sampled at 10 different places from 0–20 cm depth, using soil cores. Replicate samples were pooled together, giving 1 kg-composite samples. The samples were transported on ice and stored at −80 °C until further analysis.

To collect the bacterial cells from the different soils, soil biomass purification was conducted according to Sentchilo et al. (2013) [2]. Briefly, 15 g soil samples were homogenized by magnetic stirring for 15 min, in ice-cold poly (beta-amino) esters (PBAE) buffer (PBAE buffer is 10 mM Na-phosphate, 10 mM ascorbate, 5 mM EDTA, pH 7.0), at 10 mL g^−1^ of soil. Low speed centrifugation in 50-mL conical tubes at 160 g for 6 min was used to remove coarse particles, big eukaryotic cells and bacterial flocks. The collected supernatants were centrifuged at 10,000 *g* for 5 min to pellet the microbial biomass for further analysis.

Soil DNA was extracted using the DNeasy PowerSoil Kit (Qiagen, Germany) according to the manufacturer's instructions. DNA concentration was determined by using the Quant-iT PicoGreen dsDNA Assay Kit, and the Qubit™ 3.0 Fluorometer (Qubit, Life Technologies, USA). The three DNA samples extracted from each block were pooled together in equal nanogram quantities. Six DNA samples representative of the three cities were sent to Edinburgh Genomics for high-throughput sequencing. Sequencing was conducted using Illumina Hiseq4000 (Illumina, Inc, USA), TruSeq DNA Nano gel free library (350 bp insert) was used to prepare the libraries. Raw data consisted of 190.5 Gb sequences. The metagenomic datasets have been deposited at the National Center for Biotechnology Information (NCBI), Sequence Read Archive (SRA) under project accession number PRJNA358310.

The Short Better Representative Extract Dataset (ShortBRED) was used to identify and quantify of antibiotic resistance genes (ARGs) from the metagenomes (Kaminski et al., 2015) [3]. ShortBRED profiles protein family abundance in metagenomes in two-steps: (i) *ShortBRED-Identify* isolates representative peptide sequences (markers) for the protein families, and (ii) *ShortBRED-Quantify* maps metagenomic reads against these markers to determine the relative abundance of their corresponding families based on reads per kilobase million (RPKM). Minimum identity of 95% and minimum fragment length of 30 amino acids were considered positive. ARGs were identified with the Comprehensive Antibiotic Resistance Database (CARD) (McArthur et al., 2013) [4]. ARG markers were generated using the comprehensive and non-redundant UniProt reference clusters UniRef50 as a reference protein database. Antibiotic resistance ontology (ARO) numbers in CARD was used to aggregate, annotate and associate the ARGs to the corresponding resistance family.
